# Bonsai Gelsolin Survives Heat Induced Denaturation by Forming β-Amyloids which Leach Out Functional Monomer

**DOI:** 10.1038/s41598-018-30951-3

**Published:** 2018-08-22

**Authors:** Maulik D. Badmalia, Pankaj Sharma, Shiv Pratap Singh Yadav, Shikha Singh, Neeraj Khatri, Renu Garg, Ashish

**Affiliations:** Csir-Institute of Microbial Technology, Chandigarh, India

## Abstract

Here, we report that minimal functional gelsolin *i*.*e*. fragment 28–161 can display F-actin depolymerizing property even after heating the protein to 80 °C. Small angle X-ray scattering (SAXS) data analysis confirmed that under Ca^2+^-free conditions, 28–161 associates into monomer to dimer and tetramer, which later forms β-amyloids, but in presence of Ca^2+^, it forms dimers which proceed to non-characterizable aggregates. The dimeric association also explained the observed decrease in ellipticity in circular dichroism experiments with increase in temperature. Importantly, SAXS data based models correlated well with our crystal structure of dimeric state of 28–161. Characterization of higher order association by electron microscopy, Congo red and ThioflavinT staining assays further confirmed that only in absence of Ca^2+^ ions, heating transforms 28–161 into β-amyloids. Gel filtration and other experiments showed that β-amyloids keep leaching out the monomer, and the release rates could be enhanced by addition of L-Arg to the amyloids. F-actin depolymerization showed that addition of Ca^2+^ ions to released monomer initiated the depolymerization activity. Overall, we propose a way to compose a supramolecular assembly which releases functional protein in sustained manner which can be applied for varied potentially therapeutic interventions.

## Introduction

Plasma gelsolin (pGSN) is the most abundant actin regulating protein in our plasma, and it’s major function is to scavenge F-actin released from the damaged or dying cells, thereby protecting microvasculature of our circulatory system^1,2^. Therapeutic potential of pGSN becomes significant in cases of injuries or sepsis where the actin released in plasma rapidly polymerizes actin into its viscous filamentous form i.e. F-actin as a result of exposure to higher salt concentrations in the plasma^3^. Such F-actin is toxic to vital organs, and high levels of pGSN present in 1 mM free Ca^2+^ of plasma comes into immediate action where pGSN along with Vitamin D binding protein depolymerizes F-actin, and remains bound to disassociated actin units^2,4^. In this process, available pGSN decreases in correlation to the F-actin released in plasma or severity of the injury. It is well documented that in critical care patients, pGSN levels decrease to significantly low values and decrements below 50% of average value have been found to be associated with prolonged ICU stay, dependence on ventilator support and a significantly higher risk of morbidity^5^.

A rescue for the depleting levels of pGSN was experimented by repletion with exogenous recombinant human gelsolin (rGSN) and based on remarkable positive outcome compared to placebo; the approach has been termed as gelsolin replacement therapy (GRT)^6^. By now, GRT is well studied in case of murine and rat models challenged with burns, sepsis, hyperoxia as well as Alzheimer disease; where rGSN significantly rescues or ameliorates the symptoms of acute insults on the animals^7–9^. It is pertinent to mention here that GRT is in preclinical trials for different indicators, Phase 2 trial for Community Acquired Pneumonia (CAP), ad Phase 1 trials for Trauma and critical care, and Antibiotic Resistance (https://bioaegistherapeutics.com/pre-clinical/). The prime bottle-neck in the implementation of GRT is the overall amount of injectable rGSN required to achieve therapeutic intervention in humans. For a mouse, multiple doses of 8 mg of rGSN have been reported to be protective. Considering a simplistic linear scale-up, a single dose of about 28 g would be required for a human with body mass of 70 kg which will be a challenge both from production as well as costing terms. Bearing in mind, the promise of GRT and challenges to translate it, we opted to explore any scope of rationally minimizing the mass of the 80 kDa protein without sacrificing the required F-actin depolymerizing functionality^6^. Earlier, employing shape-function experiments, we demonstrated that first 28–161 residues of pGSN can depolymerize F-actin with relatively better kinetics than pGSN^6^. Our shape-function data showed that this bonsai version requires free Ca^2+^ ions or low pH to open up to bind actin and effect its function. Importantly, the advantage of smaller mass of 28–161 was also reflective *in vivo* as well, since the bonsai version could rescue LPS induced septic mice at doses which were four fold less than rGSN (and the latter was limiting in efficacy). Furthermore, the cytokine analysis of the LPS challenged mice treated with rGSN and bonsai gelsolin showed that the immune response of mice treated with bonsai gelsolin shifted from pro-inflammatory to anti-inflammatory^6^. All these indicate towards a possible role of the bonsai rGSN *i*.*e*. 28–161 as a substitute in the therapeutic potential of GRT.

Recently, we showed that besides free Ca^2+^ ions or low pH, physiological temperature can also activate full-length gelsolin and PIP_2_ acts as a deactivator of gelsolin^10^. While extending the experiments to other versions of gelsolin in our lab, we were surprised to observe that 28–161 could exhibit its F-actin depolymerizing activity even after being heated to 80 °C! Though we had seen Ca^2+^ ions or pH induced activation of this bonsai gelsolin, there was no information on influence of temperature on the function of this fragment of gelsolin. The rationale for this work was to understand how bonsai gelsolin can retain F-actin depolymerizing despite being treated to extreme temperatures. Detailed analysis brought forth that 28–161 has an inherent tendency to self-associate at temperatures around 30–45 °C and the association behavior is influenced by Ca^2+^ ions present in the buffer. In absence of Ca^2+^ ions, heating induces the protein to form β-amyloids, but in the presence of Ca^2+^ ions or at low pH, increase in temperature induced formation of non-specific and non-functional aggregates. Size exclusion chromatography (SEC), SAXS data analysis and biochemical assays revealed that the higher order β-amyloids keep releasing monomeric protein which upon addition Ca^2+^ ions becomes viable to bind and depolymerize F-actin. Overall, we present here a way to compose self-releasing higher order assembly of this potentially therapeutic protein which may aid in its application in scenarios requiring sustained release of this functional protein.

## Results

### F-actin depolymerization activity and stability of 28–161

**Figure 1:**
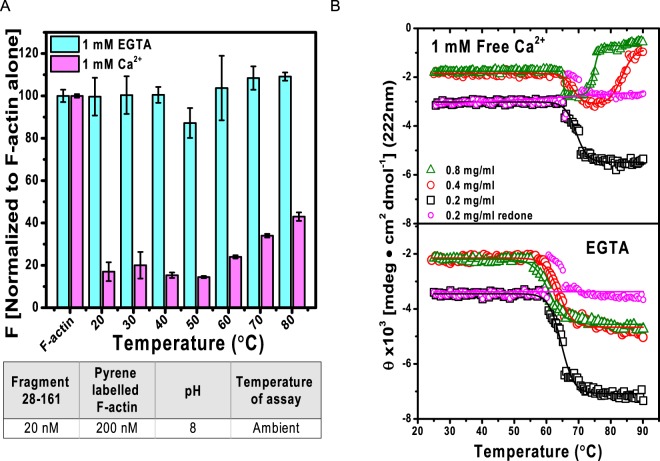
Function-shape of 28–161 as a function of temperature. (**A**) Normalized pyrene fluorescence data indicated that 28–161 protein upon heated till 80 °C followed by cooling and addition of Ca^2+^ ions somehow retained or recovered F-actin depolymerizing activity. (**B**) Variation in the MRE values recorded for 28–161 at different concentrations in presence (upper panel) and absence of Ca^2+^ ions (lower panel) are plotted here as a function of temperature.

The shape-activity relationship of 28–161 at ambient temperature has been reported earlier^6^, but its variation as a function of heat is unknown. In this work, 28–161 was heated to elevated temperatures, cooled to room temperature followed by addition of Ca^2+^ ions in sample and then the protein solution was added to pyrene-labelled F-actin. This is an established assay to evaluate ability of protein or molecule to achieve depolymerization of F-actin either by actively disassociating actin units in filament or by capping the growing ends of filaments. In some cases, depolymerization is achieved when the protein binds to and sequesters monomeric units of actin or G-actin in getting committed in F-actin formation. Results of the activity assay revealed that the fluorescence of pyrene labelled F-actin decreased in all samples of 28–161 added with Ca^2+^ ions, even after being heated till 80 °C, *albeit* with slight reduction in potency with increasing temperature (Fig. 1A). At the same time, no decrement in pyrene fluorescence was observed in samples which were not supplemented with Ca^2+^ ions (or lacking protein but having Ca^2+^ ions). This was the first observation which brought forth that 28–161 somehow retains or regains F-actin depolymerizing activity even after being exposed to extreme temperatures. Importantly, these results were significantly different from our results obtained for full length gelsolin, where in absence of Ca^2+^ ions, full-length gelsolin displayed unexpected depolymerization activity in the range of 30 to 40 °C^10^. Using SAXS data analysis and modeling, it was later deciphered to be due to selective opening of the G1 domain of gelsolin away from remaining five domains^10^. For current manuscript, repeated experiments showed that 28–161 exhibited activity even after being heated to higher temperatures, and unlike the parent molecule *i*.*e*. gelsolin, 28–161 could not be activated by temperature alone. In other words, Ca^2+^ ions are essential for activity of 28–161. To decipher the reasons for this unusual behaviour and its possible correlation with any changes in secondary structural content, we performed variable temperature CD experiments with this protein.

### Variable Temperature CD spectra analysis

Each domain of gelsolin has 3 α-helices which run across the whole domain and constitute about 17–22% of residues in that domain. Thus, in order to track temperature induced changes in the α-helical content, the ellipticity was monitored at 222 nm at different temperatures with a 28–161 solution of 0.2 mg/ml. Plot of mean residue ellipticity (MRE) computed considering monomer version of 28–161 as a function of temperature showed that there was an unexpected decrease in the negative ellipticity after 60 °C, and the decrease occurred both in samples having and lacking free Ca^2+^ ions (Fig. 1B). This indicated that there is a gain in the α-helical content in protein with increase in temperature. Interestingly, such a decrease in ellipticity at [θ]_222_ as a function of temperature has been reported earlier for α-Synuclein, and some other intrinsically disordered protein(s) which gain secondary structure when subjected to higher temperature^11,12^. To check if this gain in secondary structural content is influenced by concentration of 28–161, we acquired CD spectra at relatively increased (double) concentration of protein 28–161 *i*.*e*. at 0.4 and 0.8 mg/ml concentrations. The data collected with higher concentration of 28–161 in absence of Ca^2+^ ions showed similar gain in structural content, but in presence of Ca^2+^ ions decrease in negative intensity was observed only up to 70–80 °C after which it showed loss in α-helical content (Fig. 1B). CD data in samples having free Ca^2+^ ions indicated that the protein molecules first started associating, and then started losing helical content. It is important to mention here that in absence of Ca^2+^ ions, for the concentrations studied, the MRE values exhibited reversible nature upon decreasing the temperature from 80 °C, but samples having Ca^2+^ ions did not show any reversibility after 60 °C (*data not shown*). This observation supported that associations induced under Ca^2+^-free conditions are reversible, but associations of Ca^2+^-activated structure of 28–161 cannot revert with cooling. Simultaneously, we also monitored the HT values for each experiment and observed that though the HT values remained below 700 V in most experiments. One interesting recent report showed that sudden or abrupt increase in HT values during CD data collection can indicate occurrence of aggregation in the protein samples^13^. In our CD experiments, the HT values for 28–161 increased steadily for the protein at all concentrations studied in absence of free Ca^2+^ ions (please see Fig. S1). In presence of Ca^2+^ ions, there was a clear anomalous increase in HT values for sample having 0.4 mg/ml and slight increase in sample having 0.8 mg/ml. In addition to ellipticity values, these observations implied that there could be initiation of aggregation in 28–161 protein with increase in temperature, in presence of Ca^2+^ ions.

Presuming a two-state transition, the temperature of melting (T_m_) was calculated to be 66°, 65° and 62 °C for samples lacking Ca^2+^ ions and having concentration of 0.2, 0.4 and 0.8 mg/ml, respectively. Any case, the apparent gain in secondary structural content with increase in temperature remained intriguing. Earlier, such observations were made for disordered proteins which gain secondary structure due to heat induced reorganization^11,14^, but gain observed for 28–161 remained unclear since this protein lacks inherent disorder and demonstrates functionality at lower temperatures as well. Based on observations described below that 28–161 may form dimer, for samples at 0.2 mg/ml, we recalculated the MRE values above 65 °C by using 266 residues instead of 133 residues, for monomer. Interestingly, we found that considering onset of dimerization keeps the MRE mathematically unchanged (please see magenta symbols and fit in Fig. 1B). This processing indirectly clarified that the decrement in ellipticity values are due to association of protein, not gain in helical order with heating. In contrast, in presence of Ca^2+^ ions, samples at 0.4 and 0.8 mg/ml appeared to lose secondary structural content or denature with heating. Any case, data from our CD experiments concluded that heat induced association pattern of 28–161 and detectable changes in secondary structural content is different in presence and absence of free Ca^2+^ ions.

### Solution shape analysis using VT SAXS Datasets

**Figure 2:**
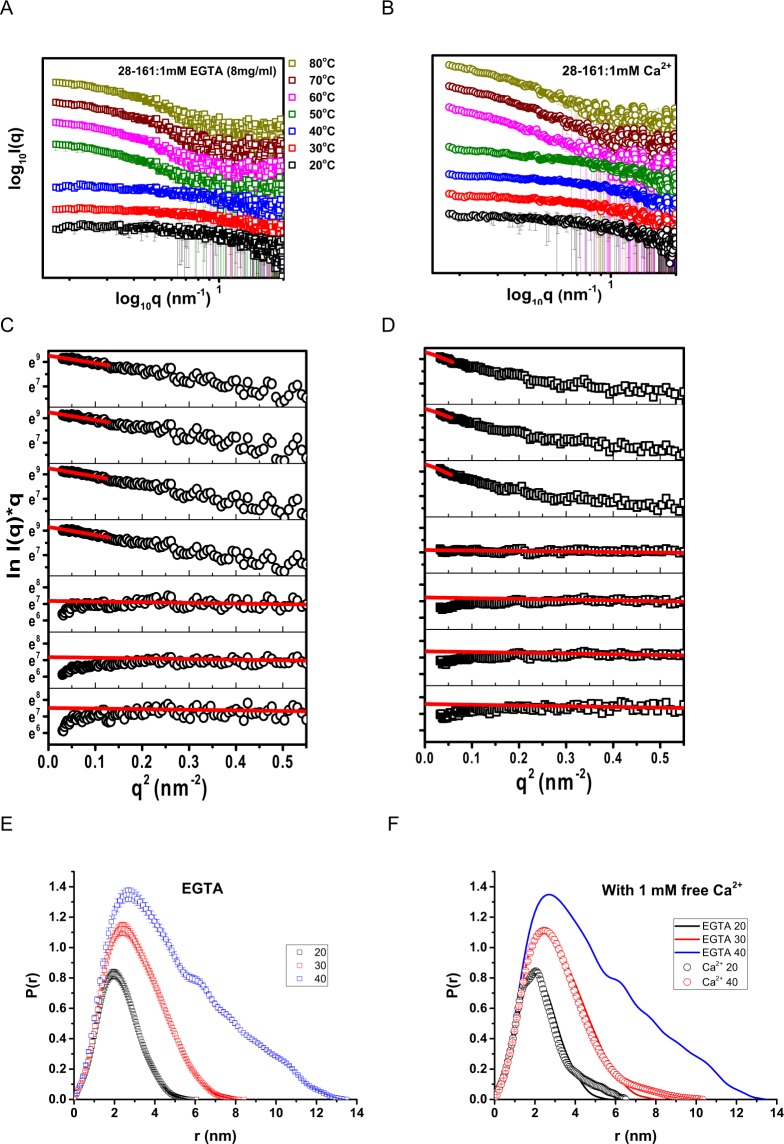
(**A**,**B**) The intensity plots of SAXS data collected as a function of temperature in absence or presence of 1 mM free Ca^2+^ ions are presented here. (**C**,**D**) Guinier analyses for rod-like shape of the SAXS datasets shown above are presented here. The red lines represent the linear range of the approximation. (**E**,**F**) The calculated P(r) curves from the SAXS datasets indicating monodisperse nature are plotted here.

Earlier, we published the SAXS data based shape parameters of 28–161 at 3.3 and 4.2 mg/ml at 10 °C in presence and absence of Ca^2+^ ions, and at low pH in absence of Ca^2+^ ions^6^. Under those conditions, especially at low temperature (close to 10 °C), 28–161 remained monomer with compact and open shape characterized by a radius of gyration (R_G_) of 16 and 18 Å, respectively. To investigate the effect of temperature on the global shape of 28–161, we collected SAXS data in presence and absence of 1 mM Ca^2+^ ions as a function of temperature. SAXS data was collected on three concentrations of 28–161 ± free Ca^2+^ ions *i*.*e*. 4, 8 and 10 mg/ml, and the shape parameters of protein molecules were similar except relatively more noise was observed in the samples at lower concentrations for same exposure times. Here, we have presented the profiles and analysis for 8 mg/ml. At every temperature, SAXS data was collected for 30 minutes before changing the temperature. The data was reduced, processed and plotted as described earlier^10^. SAXS I(Q) profiles from 28–161 at different temperatures with and without free Ca^2+^ions in buffer have been presented in Fig. 2A,B, respectively. Double log plots of I(Q) *vs*. Q supported that our samples lacked any aggregation or inter-particulate effect during data collection, but there was a clear indication of increase in the average particle size with increase in temperature seen from the steepness of the slope of intensity profiles. From the SAXS I(Q) profiles collected, it appeared that 28–161 undergoes dramatic change in its shape and/or association status between 40–50 and 50–60 °C in absence and presence of Ca^2+^ ions, respectively.

Presuming globular scattering nature of protein in solution, Guinier approximation provided R_G_ and intensity at zero scattering angles (I_0_) values for 28–161 protein at different temperatures (Table 1). The trend indicated that both in presence and absence of Ca^2+^ ions the protein was monomer at 8 mg/ml at 20 °C. With heating, the particle size immediately started increasing in absence of Ca^2+^ ions, but the incremental rate was significantly retarded in presence of Ca^2+^ ions. As mentioned above, increase in the particle size was dramatic for the protein in the temperature range of 40–50 and 50–60 °C in absence and presence of Ca^2+^ ions, respectively. Same was reflected in the estimated intensity at zero angle (I_0_) values. Using lysozyme as standard, estimated I_0_ value from Guinier analysis was used to calculate the molecular mass of the scattering species. For 28–161 in EGTA buffer or lacking free Ca^2+^ ions, computed I_0_ values indicated that the protein was monomer at 20 °C at 8 mg/ml and slowly started associating with heating (Table 1). The intensity values at 30 and 40 °C suggested that the predominant scattering species had a molecular mass of 33 and 64 kDa which supported dominant presence of dimeric and tetrameric species in solution. The next dataset acquired at 50 °C indicated presence of higher order association, and the trend remained same till 80 °C with further increase in computed dimensions. In contrast, I_0_ values computed from samples of 28–161 in buffer having 1 mM free Ca^2+^ ions indicated its existence predominantly as monomer from 20 °C to 35 °C, and the dataset acquired at 40 °C supported dominance of dimeric population in solution. With further heating, 28–161 in presence of Ca^2+^ ions transformed in to large order association from 50–60 °C.

**Table 1:** The radius of gyration (*R*_*G*_) and molecular mass (*kDa*) calculated from forward scattering intensities (*I*_0_) estimated from the Guinier region of the SAXS data are tabulated here.

Protein Temperature (°C)	*R*_*G*_ *(nm*)	Guinier Analysis		Scattering Shape from Guinier plot for rod-shape (Association state)
		*Intensity (I*_*0*_ *)*	*Calculated Mol*. *Mass (kDa)*	
Lysozyme*	1.39 ± 0.04	2390	14.2	Globular (Monomer)
*28–161* + ***1 mM EGTA (8*** ***mg/ml)***				
20	1.67 ± 0.12	22486	16.7	Globular(Monomer)
25	2.12 ± 0.11	32989	24.5	Globular (*Mixed order*)
30	2.37 ± 0.13	44703	33.2	Globular (Dimer)
35	3.27 ± 0.12	59514	44.2	Globular (*Mixed order*)
40	3.87 ± 0.09	86579	64.3	Globular (Tetramer)
50	8.79 ± 0.45	426295	316.6	Rod (Higher Order)
60	9.34 ± 0.38	814620	605.4	Rod (Higher Order)
70	8.85 ± 0.36	821352	610.1	Rod (Higher Order)
80	8.05 ± 0.37	771532	573.2	Rod (Higher Order)
***28–161*** + ***1*** ***mM free Ca***^***2+***^ ***(8*** ***mg/ml)***				
20	1.84 ± 0.11	22217	16.5	Globular (Monomer)
25	1.91 ± 0.08	22486	16.5	Globular (Mainly monomer)
30	1.89 ± 0.11	23294	17.3	Globular (Monomer)
35	2.23 ± 0.11	22210	16.5	Globular (*Monomer*)
40	2.52 ± 0.28	44703	33.2	Globular (Dimer)
50	3.74 ± 0.59	65843	48.9	Globular (*Mixed order*)
60	7.24 ± 0.55	1059410	786.8	Rod (Higher Order)
70	7.29 ± 0.60	957346	711.2	Rod (Higher Order)
80	7.58 ± 0.68	930417	691.1	Rod (Higher Order)

*Estimated for 1 mg/ml of Lysozyme for same exposure time.

N.B.: The molecular mass was calculated using this formula: $\frac{{{I}}_{{l}}}{{{m}}_{{l}}\times {{c}}_{{l}}\times {{t}}_{{l}}}=\frac{{{I}}_{{g}}}{{{m}}_{{g}}\times {{c}}_{{g}}\times {{t}}_{{g}}},$where, I = Intensity at zero angles, m = molecular mass, c = concentration, and time *t*_*l*_ = *t*_*g*_.

Modified Guinier analysis or the analysis for rod-like shapes brought forth that the protein molecules remain globular in EGTA and in presence of Ca^2+^ ions up to 40 and 50 °C, respectively (Fig. 2C,D, and Table 1). The linear fit to the modified Guinier plot with negative slope have been shown as red lines (Fig. 2C,D). In these plots, downwards “rolling-over” of points as Q → 0 nm^−1^ indicates that the shape of particle is more globular. Otherwise, alignment of data points to the linear fit supports a rod-like shape. This analysis clearly supported that 28–161 forms rod-like shapes from 50–80 and 60–80 °C in buffers lacking and having free Ca^2+^ ions, respectively. Analysis of SAXS datasets (and CD data mentioned above) implied that Ca^2+^ ions somehow regulate the shape profile of the low order association of 28–161. In summary, while 28–161 can form tetramer in solution in absence of Ca^2+^ ions, same could not be seen in presence of Ca^2+^ ions.

### Shape of the low order associated species

We could reliably compute the P(r) curves for the SAXS datasets where the calculated mass of the protein from the SAXS datasets supported predominance of monodisperse association state (Fig. 2E,F). As mentioned above, 28–161 appeared to be adopting monomeric, dimeric and tetrameric status in solution at 20, 30 and 40 °C, respectively in absence of free Ca^2+^ ions. Their P(r) curves indicated that the protein molecules in these association states adopt a solution shape characterized by a D_max_ value close to 6.1, 8.5 and 13.8 nm, and R_G_ value of 1.67, 2.37 and 3.87 nm, respectively (Fig. 2E). Similarly, the P(r) curves computed for 28–161 in presence of Ca^2+^ ions in buffer and representing monomeric (20 °C) and dimeric (40 °C) status have been plotted in Fig. 2F. Importantly, the shape parameters for the monomeric 28–161 ± Ca^2+^ ions matched with the values reported earlier by us^6^. For samples containing free Ca^2+^ ions, the monomer and dimer were characterized by an R_G_ of 1.84 and 2.52 nm, respectively which were clearly larger than the same status in absence of Ca^2+^ ions. Furthermore, comparison of the P(r) curves for the protein lacking Ca^2+^ ions indicated that there are observable changes in the frequency distribution of the interatomic vectors, but mainly in the region of longer vectors in the distribution curves. This indicated that there are shape changes in the monomeric and dimeric 28–161 influenced by the presence of Ca^2+^ ions. Furthermore, tetramer population could not be seen for 28–161 in buffer containing Ca^2+^ ions.

**Figure 3:**
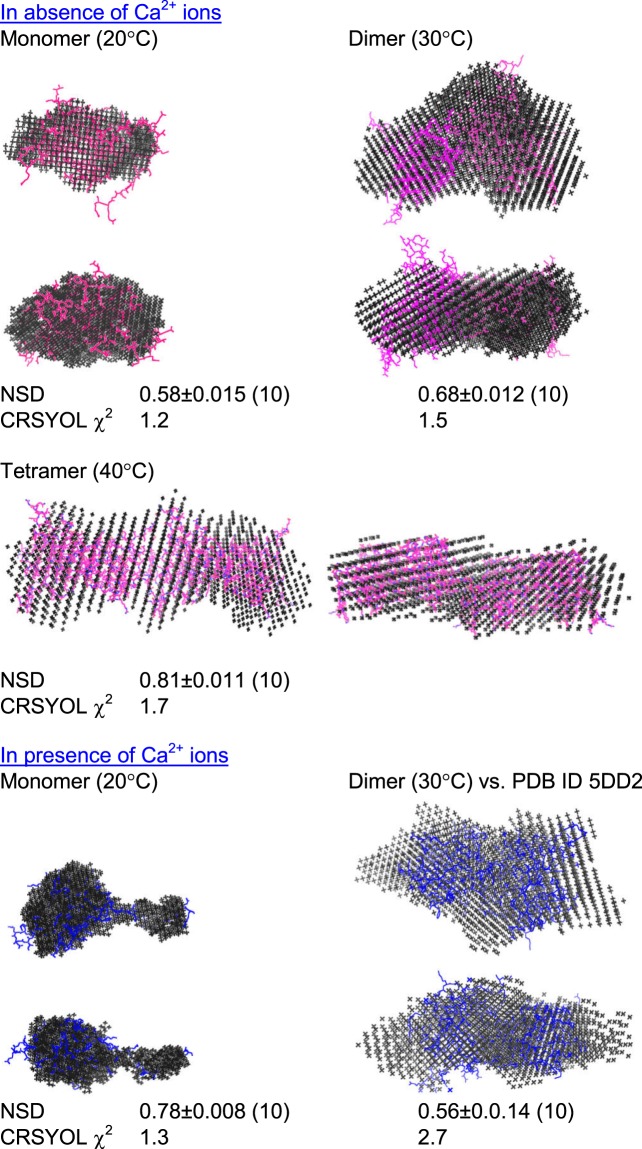
SAXS data based models are presented here. The conditions of the data collection are mentioned. SAXS data based dummy residue models are shown as grey spheres. The crystal structure or models superimposed on the SAXS data based models are shown as pink (EGTA conditions) and blue (Ca^2+^-activated) stick models. The NSD and χ^2^ values are mentioned below the models. The value in parentheses of the NSD value indicated the number of models out of 10 which were used for averaging.

Using the SAXS I(Q) and P(r) profiles, scattering shapes of 28–161 under different conditions and association state were computed and compared with available crystal or model structures (Fig. 3). For each dataset, using plug-ins integrated in the PRIMUSQT program, 10 independent runs of uniform density modeling were done, and the resultant models were superimposed by aligning their inertial axes and averaged^15,16^. Finally, the averaged model was optimized to better represent the experimental data. The normalized spatial disposition (NSD) values and their variance, a measure of similarity of the shapes of individual models, have been mentioned below the models (Fig. 3). In this work, all the ten models calculated for each system were within the range for averaging. In absence of Ca^2+^ ions, the model of the monomer of 28–161 compared well with the model of same sequence abstracted from the crystal structure of inactive or Ca^2+^-free full length gelsolin (PDB ID 3FFN). The model for the dimer could accommodate two chains of 28–161 and similarly, four chains of 28–161 could be superimposed with the shape volume of the model for the tetramer of protein. *The residue models of the associated states of inactive 28–161 were generated as described below using the packing relationship observed in our crystal structure PDB ID 5ZZ*0. Similarly, the model solved for the monomer of 28–161 in presence of Ca^2+^ superimposed with the structure of G1 domain abstracted from the structure of first half of gelsolin bound to actin showed an extended g1-g2 linker. Importantly, the shapes of monomers of 28–161 ± Ca^2+^ ions compared well with our previous report^6^. The shape model solved for the dimer of the protein at 30 °C could accommodate two chains of activated 28–161 inside as solved by crystallography by us (*described below*).

**Figure 4:**
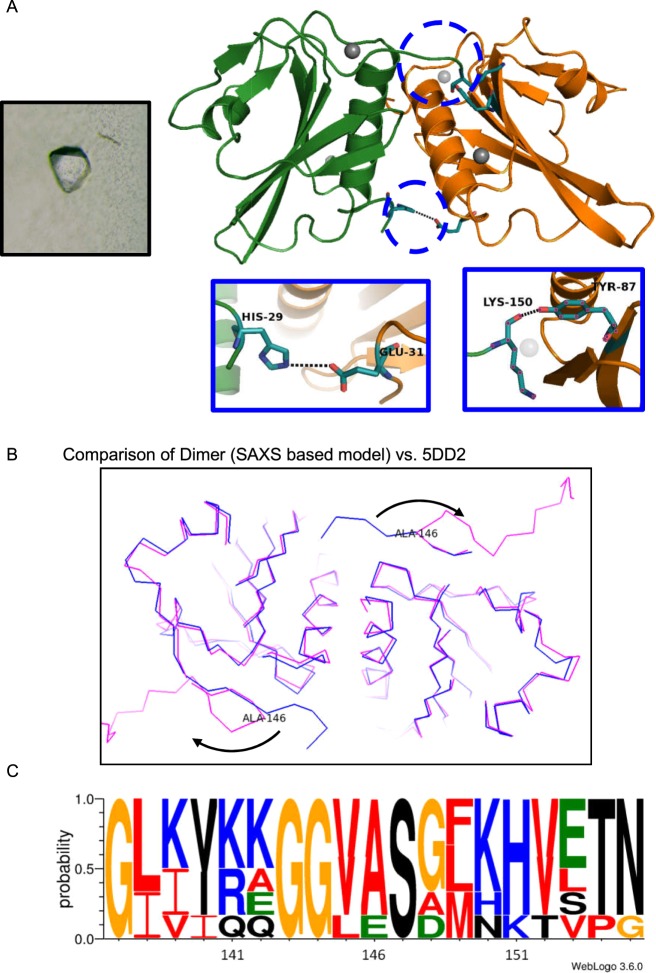
Crystal structure of the dimer of 28–161 in presence of Ca^2+^ ions is presented here. (**A**) The image crystal used for diffraction is shown as left inset. The refined crystal structure is shown as ribbons (PDB ID5ZZ0). Specific interactions which contribute most to the interface are highlighted. (**B**) Comparison of the model built for dimer of 28–161 under Ca^2+^-free condition and compared with SAXS data (and based model) is shown here. The superimposed C^α^ trace highlights that the Ala146 acts as a fulcrum of repositioning of the tail region upon binding to Ca^2+^ ions. (**C**) The logo highlights the conservation of residues at different positions in sequence of the different gelsolin family proteins.

In absence of any crystal structure of 28–161 as monomer or in associated state for comparison with SAXS data based models, we attempted to obtain diffractable crystals from the protein as a function of concentration of protein, presence or absence of Ca^2+^ ions in buffer and employed range of elevated temperatures. Of the different conditions tried, we could obtain diffractable crystals only at 18 °C and in the presence of Ca^2+^ ions (Fig. 4A). Refinement of the diffraction data brought forth that there were two chains of 28–161 in the asymmetric unit (PDB ID 5ZZ0; Table 2). Rotated views of the electron density maps at 2F_0_ − F_C_ levels with refined chains of 28–161 and their arrangement of different chains in the unit cell are shown in Fig. S2. PDBePISA analysis^17^ computed interface surface area to be less than 700 Å^2^ for the best possible combination of two chains in the symmetry (Table S1). This indicated that as per crystal packing profile, 28–161 may not form dimer. Furthermore, the computed complex formation significance score (CSS) for the interfaces scored 0 which supported that the interface visualized in the crystal packing does not support complex formation. This differential conclusion from the confirmed observation of dimeric association from SAXS data analysis can be interpreted as: 1) the crystal was obtained at 18 °C and may not truly represent associations occurring at higher temperatures, and/or 2) weak interactions between native monomeric structures seed the beginning of higher order associations, as seen before^18^.

**Table 2:** Data collection statistics from the crystal of fragment 28–161 in presence of Ca^2+^ and at low pH are tabulated here. Values in the parentheses refer to the highest resolution shell.

**Data collection**	
Resolution (Å)	50–2.64
Space Group	P2_1_ 2_1_ 2_1_
Unique Reflections	6377
Unit cell parameters	
a (Å)	35.39
b (Å)	63.57
c (Å)	98.14
α (°)	90
β (°)	90
γ (°)	90
Completeness (%)	91.6 (67.1)
R_merge_	0.11 (0.56)
Multiplicity	4.9 (3.1)
Average I/σ (I)	14 (1.65)
**Refinement**	
R_work_ (%)	21.57
R_free_ (%)	25.44
Solvent Content	32.57
Chains	G, A
R.M.S. deviation from ideality	
Bonds (Å)	0.010
Angles (°)	1.37
Wilson B factor (Å^2^)	47.93
Ramachandran plot statistics (%)	
Most favoured	86.3
Allowed region	13.2
Generously allowed	0
Disallowed	0.5
PDB code	**5ZZ0**

Any case, our crystal structure showed two Ca^2+^ ions bound to each chain of 28–161 and the G1 domains were arranged next to each other with few interactions stabilizing the interfacial contact (Table 3). Each chain showed the typical gelsolin domain architecture of five β-sheets sandwiched between the two α-helices, with long helix running parallel to the β-sheets whereas the small helix was perpendicular to the β-sheets. Importantly, during refinement, we could not identify any electron density corresponding to the g1-g2 linker, possibly due to inherent flexibility across these residues. A similar observation was reported in a recent multi-dimensional NMR experiments based study where 28–161 was studied at two pH values, 7.3 and 5 and no nuclear Overhauser effects (NOEs) could be recorded for the residues 145–161 under either condition. This was attributed to the highly disordered nature of these residues, and thus the orientation of the tail could not be defined^19^. In our crystal structure, four Ca^2+^ ions were present in the entire assembly, two bound to each monomer, including both Type I (G^65^, E^97^, V^145^) and Type II (D^109^, G^114^, A^116^) Ca^2+^ binding sites. The interaction analysis revealed that the dimer is stabilized by one classical hydrogen bond between K150 → Y87, a salt bridge between H29 → E21 and numerous weak non-bonded interactions (Table 3)^20^. Since all crystals of this portion of gelsolin have been solved bound to actin, we also compared the interactions between the two chains with that of G1 domain of gelsolin and actin. Alignment of C^α^ trace of one chain in our crystal structure with same residues in the crystal structure of Ca^2+^-activated N-terminal half bound to actin provided an RMSD value of 0.45 Å. This information support that Ca^2+^ ions are sufficient to induce changes in G1 domain to bind actin and no additional significant changes happens post-binding to actin. Interestingly, using the G1 domain- G1 domain relative orientation in space seen in our crystal structure (PDB ID 5ZZ0) and using the coordinates of 28–161 from inactive gelsolin crystal, we generated a putative model of dimer of 28–161 in absence of Ca^2+^ ions. This model fitted very well inside the SAXS data based envelope model solved at 30 °C (Fig. 3). A computed χ^2^ value of 1.5 between the calculated SAXS profile of this model and experimental SAXS I(Q) profile (between Q range of 0.07–0.5 nm^−1^) supported modelled structure of inactive dimer. Further, the symmetry mates of crystal structure were used to generate different possibilities of tetramer of inactive 28–161. Using the SAXS model as a filter, only one option remained and it fitted with the SAXS data based envelope model and computed *vs*. experimental SAXS profile supporting the model of tetramer thus generated. This indicated that the formation of tetramer from dimer of 28–161 does not involve substantial structural rearrangements in the individual chains and relative orientation. Comparison of the two states of dimer of 28–161, one seen from our crystal structure bound to Ca^2+^ ions and other model fitted inside SAXS data based envelope to represent Ca^2+^-free dimer state showed that the difference lies in the relative orientation of the g1-g2 linker (Fig. 4B). Of particular interest is the residue Ala146 which acts as a fulcrum of this differential positioning with almost 180° flip of the tail region after it. To validate if Ala146 is critical for this difference between 28–161 in presence and absence of Ca^2+^ ions, we first analyzed its conservation in the gelsolin family of proteins. The alignment shown in Fig. 4C brought out that A146 is highly conserved and positioned close to fully conserved G143 and G144, and highly conserved V145 and fully conserved S147. This indicated that this short segment is constitutionally specific and possibly important for the Ca^2+^-activated functioning of gelsolin. We attempted to characterize A146G, A146L and A146P, but to date none could be expressed. Till now, our results imply that the flipped or open positioning of the g1-g2 linker somehow interferes with a distinct observation of tetrameric state of 28–161 and/or promotes higher order association, faster than the changes occurring in absence of Ca^2+^ ions.

**Table 3:** Table of residues involved in the interactions between weakly associated dimer of fragment 28–161. The highlighted resides (underlined) are the residues that are also involved in interaction with actin.

Hydrogen bonding	Salt Bridges	Non-bonded contacts
Chain A – Chain G	Chain A – Chain G	Chain A – Chain G
Lys 150 – Tyr 87	His29 – Glu31	Phe49 – Ala102
		Val106 – Phe49
		Ile103 – Phe49
		Ile103 – Asp96
		Ile103 – Ile103
		Gln107 – Ile103
		Gln107 – Gln107
		His29 – Gln107
		Pro30 – Gln107
		Asp50 – Asp110
		Lys48 – Val106
		Gln95 – Gln95
		Lys150 – Arg120
		Lys 150 – Gln118

The interactions were generated using EBI PDBSUM^20^ and the interactions with actin were determined from the crystal structure 1P8Z.

### Characterization of higher order association of 28–161 ± Ca^2+^ ions formed upon heating

**Figure 5:**
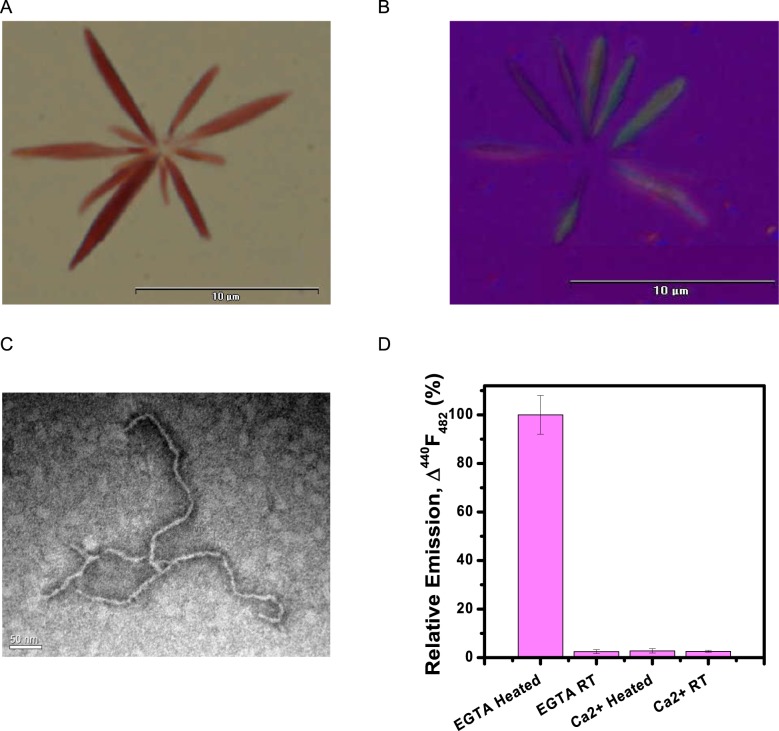
Characterization of heat induced amyloid formation of 28–161 under Ca^2+^ free conditions. (**A**) Image taken under a light microscope, at 2000X showing pinkish red Congo red stained amyloid fibrils. (**B**) Image captured under cross polarizer confirming apple green birefringence characteristic of β-amyloidic nature of fibrils. (**C**) Image captured by TEM which showed long fibrils with a uniform width of 5–7 nm. (**D**) Relative emission observed in the ThT assay for different samples have been plotted here.

As detailed above, SAXS data analysis confirmed that upon heating 28–161 formed low order association species which further associated into higher order rod-like shapes. Careful observation of samples after SAXS experiments showed whitish aggregation in samples having free Ca^2+^ and a clear gel like viscous nature of samples lacking free Ca^2+^ ions. We had encountered similar nature of samples when forming heat induced β-amyloids of globular protein lysozyme, as well as in case of transformation of G-actin into F-actin^18^. To probe the inner architecture of these rod-like associated species in both presence and absence of Ca^2+^ ions, we performed Congo red birefringence assay, a classic methodology to confirm the presence of cross-β-sheet architecture or β-amyloidic nature of samples. Samples of 28–161 were heated at same concentration in presence and absence of Ca^2+^ ions in a thermal cycle from 10 to 80 °C. Post staining with Congo red, samples were examined under microscope with light polarizer aligned and at 90° to each other. Only the samples heated in absence of Ca^2+^ ions showed the classic pink red particles with characteristic apple green birefringence upon changing the alignment of polarizers (Fig. 5A,B). This was the first confirmation that the rod-like particles seen in SAXS were β-amyloidic assemblies upon heating in buffer free of any Ca^2+^ ions. Findings further corroborated with TEM analysis of samples where fibrils of ∼500 nm in length and width ranging from 5–7 nm were observed (Fig. 5C). Guinier analysis for rod-like particles computed cross-sectional radius (R_C_) of 3.2–3.7 nm of the amyloids of 28–161 which agreed with TEM analysis. Additionally, following this process, β-amyloids were prepared with 6 and 11 mg/ml protein and studied with no differential changes in morphology of the fibrils, except that the fibrils synthesized under higher concentration were densely packed as compared to 6 or 8 mg/ml. To further confirm the amyloidic nature of the fibril, we further performed the ThT fluorescence assay of the samples.

Thioflavin T (ThT) fluorescence assay is a spectroscopic assay where the change in the fluorescence intensity of ThT (at 482 nm) is significantly enhanced upon binding to amyloid fibrils^21^. Figure 5D shows the emission of the ThT dye added to samples with heated and unheated 28–161 relative to the absorbance seen in blank (or buffer). Considering, an average value of three experiments from heated samples to be 100%, intensity observed for unheated 28–161 protein was merely 2.5%. No such observation of increase in emission value was observed with samples of 28–161 with Ca^2+^ ions in buffer; either heated or unheated (Fig. 5D). In summary, the Congo red and ThT staining experiments confirmed that the higher order associated states of 28–161 in absence of Ca^2+^ ions are β-amyloids. It still left the primary question unanswered: *how 28–161 is able to show F-actin depolymerizing activity even after heating?*

### SEC Elution profile of heated protein 28–161

**Figure 6:**
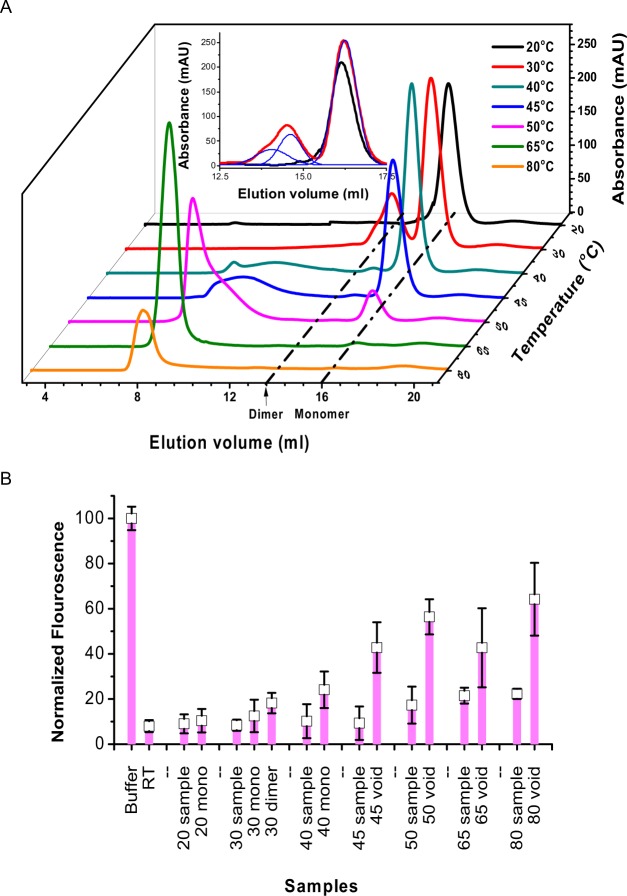
(**A**) SEC elution profiles of the samples heated at different temperature are presented here. Inset shows the comparison of the peaks seen for the samples at 20 and 30 °C. The latter shows additional peaks corresponding to mass of dimer of 28–161. (**B**) The F-actin depolymerization activity of the heated samples and different peaks collected from their SEC are shown here. The samples or fractions for which activity are shown have been mentioned below.

To address the above question, we wondered if lower order associations or monomeric form of 28–161 can release from β-amyloids upon bringing the heated samples back to room temperature? To confirm this, we performed series of SEC elution experiments with protein at different temperatures in absence of Ca^2+^ ions. For this experiment, 250 μL of protein at 8 mg/ml in EGTA buffer was heated at different temperatures for 30 minutes, followed by cooling to room temperature. Then 200 μl of the sample was injected on SEC (maintained at room temperature), and their elution profiles are shown in Fig. 6A. Sample kept at 20 °C showed a single peak close to 16 ml which corresponded to mass of 17–18 kDa confirming previous data that 28–161 remained monomer. Interestingly, the sample treated to 30 °C showed an additional peak at 13 ml suggesting a molecular mass of 35–36 kDa implying dimer of 28–161. A closer view of this new peak at 13 ml indicated that it could be composed of two species (please see deconvulation shown in the inset of Fig. 6A). This observation correlated with our SAXS based findings. Upon increase in temperature to which the samples were subjected, broader humps possibly arising from various orders of association were seen with increase in peak at 8 ml, corresponding to the void volume of the SEC set-up. This indicated that treatment of higher temperatures converted most of the protein in higher order associated species, but interestingly, elution profile up to 50 °C showed that there was presence of peak corresponding to monomer. This data implied that monomeric entity exists in equilibrium with higher order state or vice versa.

Each of the sample and collectable peaks were tested for its F-actin depolymerization activity (Fig. 6B). Please note that no sample concentration step was performed as it would have altered the association status, and depolymerization experiments were done within 5 minutes of fraction collection. Also note that no depolymerization activity was observed any sample or fraction without addition of Ca^2+^ ions which confirmed that the F-actin binding active state is induced by binding to Ca^2+^ ions. Repeated experiments showed that samples kept at room temperature or not subjected to heating, and monomeric peak from gel filtration showed expected F-actin depolymerization. Activity was comparable in the peak corresponding to dimeric protein post-treatment to 30 °C. All fractions corresponding to monomeric fraction showed activity, and so did the overall sample which was injected into the column. Interestingly, the depolymerization activity appeared to decrease with fractions corresponding to void peak, and there was a clear difference in observed activity between the same overall sample(s) before injecting into column (please see activity profiles for 28–161 treated to 45, 50, 65 and 80 °C in Fig. 6B). Latter suggested that the overall sample has some active fraction which is not eluted as a clear observable peak or peaks in the SEC elution profile. Also, samples heated and then stored at room temperature, with increasing time their peak at void volume decreased with clear increase under the hump. *These results support that the β-amyloidic assembly formed upon heating of 28–161 may not be very stable*, *and monomeric protein possibly leaches out from this supramolecular association*. *Finally*, *addition of Ca*^*2+*^ *ions activate this monomeric protein to bind and depolymerize F-actin*.

### Effect of osmolytes on sustained release of 28–161 from β-amyloids

**Figure 7:**
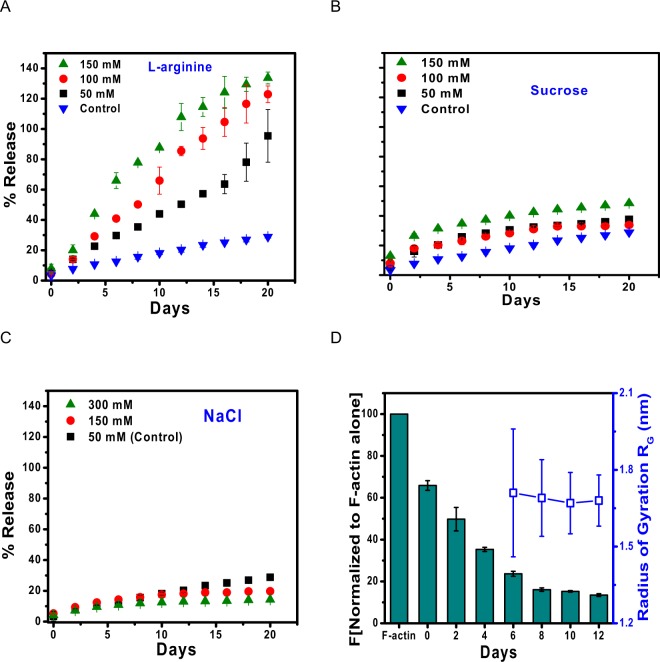
(**A**–**C**) Release profile of soluble versions of 28–161 from heat induced β-amyloids are presented here in presence of varying amounts of L-arginine or Sucrose or NaCl. (**D**) The F-actin depolymerization activity of the supernatant released in presence of 50 mM of L-Arg and sample’s R_G_ value as characterized by Guinier analysis of 20X concentrated supernatant are shown.

Osmolytes are known to de- and stabilize protein assemblies in solution, particularly when globular proteins transform into on-pathway assemblies^18^. Solution of 8 mg/ml of 28–161 was heated to 80 °C for 30 min and allowed to cool to room temperature. At regular intervals of time, the sample was subjected to quick centrifugation, and a small portion of supernatant was taken out for measurement of absorption at 280 nm to estimate protein content. Due care was taken to supplement the sample with buffer, correct for dilution and sample was mixed by vortex and kept aside till further fraction collection (Fig. 7). To observe any effect of osmolytes, different concentrations of L-arginine or sucrose or NaCl were tried by re-suspending heat induced β-amyloids of 28–161 in the buffer A containing these osmolytes. The absorbance values were used to collect percentage release of soluble protein in supernatant from the β-amyloidic assembly which settle during centrifuge. Data analysis showed that in our comparative experiments, L-Arg induces release of soluble versions of 28–161 (Fig. 7A) and the effect was significantly pronounced than sucrose or NaCl (Fig. 7B,C, respectively). In presence of 150 mM of L-Arg, almost all the 28–161 present in heat induced β-amyloidic supramolecular assembly released by 15 days (Fig. 7A).

To characterize the soluble versions getting released in solution, we prepared another three aliquots of heat induced β-amyloids as described above. To induce release, we added only 50 mM L-Arg in resuspension buffer. As done in experiments mentioned above, supernatant was collected at different days, and was analyzed by SDS- and native PAGE, and F-actin depolymerizing activity, without any protein-concentration step. Migration pattern on both PAGE experiments showed feeble amount of protein close to unheated sample indicated release of 28–161 from β-amyloids up to 4^th^ day (data not shown). Importantly, β-amyloids are known to be resistant to protein degradation and our gels confirmed no degradation of 28–161 occurred during storage at room temperature. F-actin depolymerization assay showed that the released protein could achieve depolymerization after addition of Ca^2+^ ions (Fig. 7D). Additionally, samples obtained from 6^th^ day onwards were concentrated 20X times, and their SAXS profiles were collected. Guinier and other analysis confirmed that the protein molecules were having R_G_ close 1.67 nm and other parameters close to inactive 28–161, respectively (Fig. 7D). Overall, our experiments obtained data that monomeric 28–161 release from β-amyloids which can be functionalized by Ca^2+^ ions.

## Discussion

Bonsai gelsolin or fragment 28–161 of gelsolin represents a single domain of gelsolin family of proteins with slight tail or portion of g1-g2 linker of gelsolin which allows it to bind actin. For this step, extension of the tail is necessary which is achieved upon binding to Ca^2+^ ions or exposure to low pH. Little shape-function work has been performed on this domain alone though it can depolymerize F-actin more efficiently than native protein i.e. gelsolin^6,19^. While exploring thermal stability of gelsolin we recently delineated that gelsolin can also be activated by physiological temperature^10^. Extending the work to truncated versions of gelsolin capable of depolymerizing F-actin, we observed that bonsai gelsolin could display F-actin depolymerizing ability despite being heated to high temperatures. To understand the reasons enabling this unique property to 28–161, we executed experiments described in this work. Results summarized in Fig. 1A confirmed that provision of free Ca^2+^ ions is essential by the heated samples of 28–161 to exhibit F-actin depolymerization activity. CD data implied that dimers of 28–161 can form during heating (Fig. 1B). Subsequently, SAXS data collected for samples with higher concentrations supported formation of dimer and tetramer, and only dimers could be observed for samples heated in absence and presence of Ca^2+^ ions, respectively (Figs 2 and 3). While SAXS data based dummy residue models provided cues on the envelope shapes of associated 28–161 in solution, our crystal structure of dimeric Ca^2+^-bound 28–161 helped in obtaining residue level information into dimers and tetramers both in presence and absence of Ca^2+^ ions (Figs 3 and 4). Good correlation between solution shapes and symmetry generated dimers and tetramers of 28–161 support that the relative positioning of protein chains in crystal space reliably represent the arrangement of heating induced association happening in solution. Prior to us most crystallography based structural studies of this fragment have been done in complex with actin. Interestingly, these interactions are common with the interactions formed between G1 domain and actin (Table 3). The dimerization of 28–161 seen in our structure is supported by hydrophobic interactions involving 100AAAIF104, V105 and L107. Earlier, it has been postulated that loss of hydration shell upon heating is compensated by hydrophobic interactions which results in association of native-like shapes of primary globular protein^14,18^. Support for this proposition could be seen in our earlier work where we showed that heating induces dimeric and trimeric association of native-like structures of lysozyme which turned out to be on pathway precursors of non-reversible β-amyloids^18^. Small molecules, L-arginine and benzyl alcohol which disrupted the inerfacial interactions also altered the large scale morphology of crystals and the amyloid formation profile of lysozyme. With that work, we hypothesized that native like asociation of structures precede large order association leading to β-amyloid formation, especially a large scale collective redistribution of secondary structure occurs towards cross-β-sheet formation^18^. As per literature, after lysozyme, this is probably the second example where variable temperature SAXS in conjection with protein crystallography has been applied to decipher shapes of heat induced early states of association of proteins.

**Figure 8:**
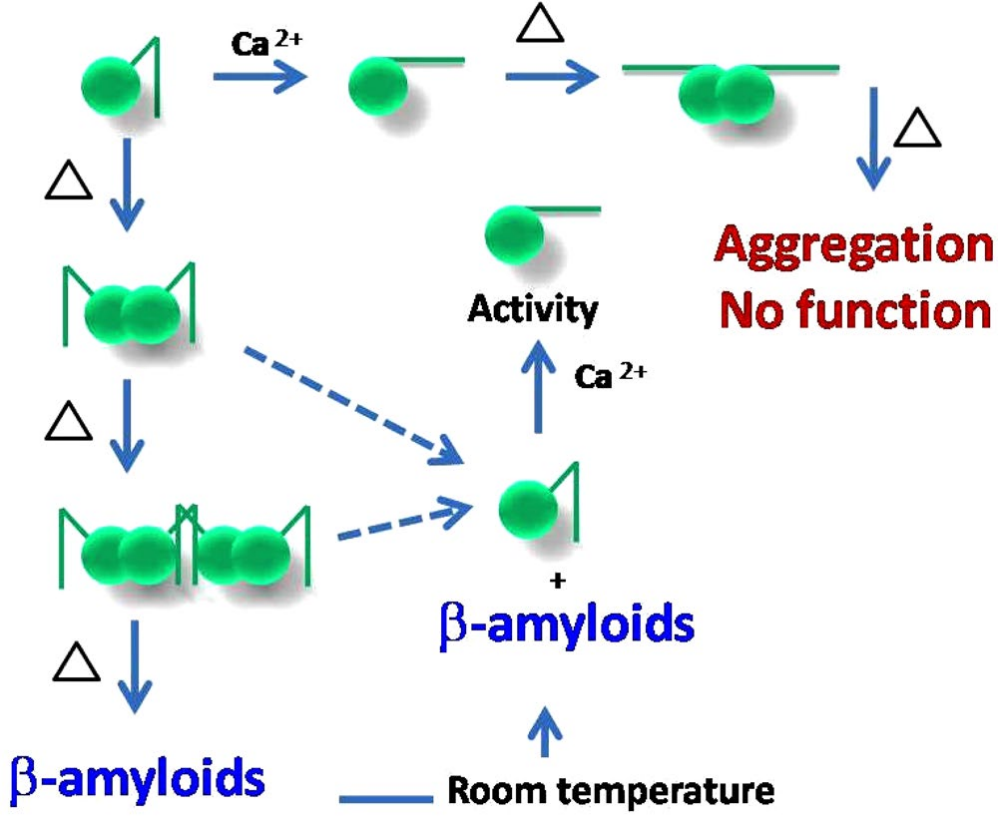
This schematic representation depicts the monomer-dimer/tetramer and oligomers of 28–161 as a function of temperature and Ca^2+^ ion shown here summarizes the results observed in this work.

We found that the tetrameric association of 28–161 formed under Ca^2+^-free conditions acts as building block for the higher order β-amyloidic association. In contrast, in presence of Ca^2+^, the initial dimer packs into higher order association is mere aggregation (Figs 4 and 5). Amyloid formation by fragments of mutant gelsolin and their role in familial amyloidosis of Finnish type (FAF) have been reviewed^22^. Briefly, autosomal mutations at D187 in the G2 domain of gelsolin *i*.*e*. D187N or D187Y compromise its ability to bind Ca^2+^ ion leading to its degradation by furin in the Golgi bodies. Resulting 68 kDa fragment of gelsolin undergoes further endoproteolysis events mediated by matrix metalloprotease leading to smaller 5 and 8 kDa fragments which form and deposit as amyloids. To intervene the amyloid formation by actual disease-associated gelsolin fragment, of the different small molecules attempted, water soluble polylactic co-glycolic acid (PLGA)-encapsulated emetine was found to defibrillate pre-formed gelsolin amyloids^23^. Even, a nanobody, Nb11 capable of tightly binding to G2 domain of gelsolin can shield it from aberrant proteolysis and represents a novel strategy in gelsolin-amyloid induced diseases^24^. Additionally, three nanobodies FAF (Nb1–3) raised against the 8-kDa fragment have been found to bind gelsolin amyloids and the concept is being developed as non-invasive imaging of amyloid deposits in mouse model^25^. Recently, we showed that under extended incubation at low pH, evn the full-length protein, pathogenic D187N mutant of gelsolin forms β-amyloids^26^. Thus, there are reports of aberrations in G2 domain of gelsolin leading to amyloids of its smaller fragments and protein as a whole, but this is the first report where isolated G1 domain of gelsolin can form amyloids by heating under Ca^2+^ free conditions. Though parallels in the overall fold of G1 and G2 domains and similarity in the constituent residues can be drawn, data shown in Figs 6 and 7 supported reversible nature of the amyloids formed by 28–161 which remains unique to date. These results and the relationship between various association states of 28–161 observed upon heating have been summarized in Fig. 8. Overall, we think our work represents a case example where under certain conditions “functional amyloids” can be formed which leach out functional or activation-competent monomeric protein. Another good example is the controlled formation of supramolecular assembly of insulin which releases functional insulin in sustained manner^27^. In that work, inherent property of insulin to aggregate towards amyloids was regulated in a manner that the assembly released insulin for extended periods. This reduced multiple doses of insulin to a single dose of assembly and managed blood glucose levels for extended periods. In our work, the controlled formation of supramolecular process involves heating 28–161 in absence of Ca^2+^ ions, and adding L-Arg or other osmolytes to the preparation to regulate release rates of functional monomer.

Levels of gelsolin has been reviewed to decrease in myriad diseases, particularly so for the critical care conditions, and in animal models, repletion with recombinant form significantly improves the outcome compared to placebo^5^. As of today, repletion is under clinical trial for various health indicators, and the concept is termed as GRT. One bottleneck in this translation is the high doses to be given to buffer out the decreased levels of plasma gelsolin due to disease or disorder. To gain advantage in the ratio of biomass input to output via fermentation methods, we reported therapeutic potential of minimized gelsolin, 28–161^6^. In biochemical assays and in animal model of LPS-induced sepsis, this molecule exhibited better rescue profile than parent molecule, offering it as an alternative approach to GRT. Our current work is an observation which can convert this bonsai protein version of a therapeutic protein into supramolecular assemblies which can release functional protein as a function of time. As demonstrated for insulin^27^, we feel that our work will inspire other researchers to translate these findings into formulating: 1) injectable amyloidic concoctions which will release F-actin depolymerizing version, and/or 2) topically applicable gels of amyloidic state which will release functional protein for cosmetic/transdermal applications.

## Materials and Methods

### Protein Expression and Purification

Bonsai version of gelsolin or fragment 28–161 was expressed in *E*. *coli* and purified as described earlier^6^. Briefly, the protein was expressed using IPTG induction in *E*. *coli* cells harbouring plasmids containing gene for C-terminal His tagged fragment 28–161. The protein was purified to homogeneity using immobilized metal ion affinity chromatography. The purity of the eluted was confirmed using 15% SDS-PAGE. The protein was further purified through FPLC, using a Superdex 200 10/300 column (GE Healthcare Bio-Sciences AB, Sweden) connected to a ÄKTA purifier (GE Healthcare Bio-Sciences AB, Sweden). The FPLC purified protein was concentrated and stored in buffer A (50 mM Tris-Cl, 50 mM NaCl, 1 mM EGTA, pH 8) at −20 °C. The concentration of protein was measured using absorbance value at 280 nm and calculated molar extinction coefficient of 21430 M^−1^ cm^−1^ (%A = 1.271). The protein was thawed in ice, and used as and when required.

### Pyrene Labelled F-Actin Depolymerization

As reported earlier, the rate of decrement in the fluorescence of pyrene-labelled F-actin in presence of heated fragment 28–161 was used to assess functionality of the protein by its ability to depolymerize F-actin^6,10^. Briefly, the protein was heated at temperature range 20 to 80 °C and cooled to room temperature. The depolymerization data was collected for the heated protein in presence as well as absence of 1 mM Ca^2+^ ions. When required, to ensure absence of Ca^2+^ ions, excess EGTA (final concentration close to 1 mM) was added to those samples. Pre-heated 28–161 was mixed with Pyrene labelled F-actin in 1:10 ratio in 96 well flat bottom FluoroNunc plates. F-actin was added last in the wells and readings were taken within 15–20 seconds of final addition. Measurements were recorded with excitation wavelength at 365 nm and emission was recorded at 407 nm using a Tecan plate reader with i-control software (Mannedorf, Switzerland). Data reported here is an average reading of three independent runs.

### Circular Dichroism (CD) measurements

To study the change in the secondary structural content of 28–161 as a function of temperature and Ca^2+^ ions, CD experiments were performed on a JASCO spectrophotometer 815 (Tokyo, Japan). A quartz cell of 0.2 cm path length was used to collect temperature scans in the range of 25 to 80 °C, with a ramp of 3 °C/min. The temperature of the cell was controlled with a Peltier type temperature control system, model PTC-42S. The ellipticity recorded at 222 nm were plotted to deduce melting temperature of protein, T_m_ which represents the midpoint of transition when half population of the protein existed in the two presumed structural states.

### SAXS Data Collection, Processing and Analysis

All SAXS data for this study were acquired on the in-house SAXSpace instrument (Anton Paar GmbH, Graz, Austria) on a line collimation beam arising from a sealed X-ray tube. Scattering data was collected on a 1D CMOS Mythen detector (Dectris, Switzerland). The data collection and processing was done exactly as described earlier^10^. Briefly, the data subtracted for buffer composition and desmeared to represent true point collimation was analyzed using different plugins in PRIMUSQT program. Ten different models were made for each dataset using DAMMIF program, superimposed, averaged using DAMAVER programs and re-optimized using DAMMIN program. Superimposition with crystal and modelled structures were done using ATSAS plugin for PyMol program. All representations were made using PyMol program.

### Heat induced amyloid formation

For heat induced amyloid formation of fragment 28–161, the protein was slowly heated in a MyGeneTM Series Peltier Thermal cycler (LongGene Scientific Instruments Shanghai, China). 50 μL of 8 mg/ml protein fragment 28–161 stored in buffer A was heated from 10 to 80 °C. The protein was heated slowly, with holding time of 30 minutes with every 10 °C increase in temperature. Post heating the samples were used immediately or stored at room temperature (~25 °C).

### Congo red birefringence assay

This is an assay developed for confirming presence of β-amyloidic structure in samples where the samples are stained with Congo Red dye and birefringence phenomenon is studied under a microscope equipped with polarizers. When the polarizers are aligned, the stained sample appear as reddish pink, but if the polarizers are aligned at 90° angle to each other, the background turns black and presence of apple green birefringence in the sample is considered to be positive test for presence of amyloids in the sample^21,28^. Congo Red staining was performed as described earlier^18,21^. Essentially, saturating amount of NaCl was dissolved in 80% ethanol: 20% Double Deionized water solution. This solution was filtered to remove any undissolved NaCl and saturating amount of Congo Red was added this solution by stirring. The final solution was filtered again using a 0.22 μm syringe filter to obtain the working solution for Congo Red Birefringence assay. 10 μl of protein sample was loaded on a clean grease free glass slide and was allowed to air dry, this sample was then flooded with Congo Red dye prepared above. The sample was covered with dye for nearly a minute and excess dye was blotted away using lint-free paper tissue, and the slide was allowed to dry at room temperature. The slides were then examined under polarization microscope for apple green birefringence (BX-51, Olympus).

### Thioflavin T (ThT) assay

The binding of ThT to amyloids results in a characteristic hyperchromic fluorescence excitation shift which can be measured by spectroscopic assay. This assay was performed as described earlier, where 4 mg ThT was dissolved in 5 ml of phosphate buffer (10 mM phosphate, 150 mM NaCl, pH 7.0), the resulting solution was filtered through 0.2 μm syringe filter and stored in dark as stock solution^21^. For performing the assay, ThT stock solution was diluted 50X in phosphate buffer to make a working solution. For measurement, the vial containing amyloids was rigorously mixed by vortex followed by centrifugation at 5000 rpm for 5 minutes, and the supernatant was used for staining and analysis. 10 μl of protein sample was mixed with 1 ml of working solution of ThT. For β-amyloids, binding of ThT dye to amyloids induces a characteristic hyperchromic fluorescence excitation shift or substantial increase in the intensity of the emission spectra in the range of 470–490 nm with peak at 482 nm. Data was recorded with an averaging over 60 seconds using a PTI Fluorimeter^29^. Comparable amount of untreated protein and buffer were used as a negative control and blank, respectively.

### Transmission Electron Microscopy (TEM)

Amyloids of 28–161 formed by the above mentioned protocol were analyzed by TEM. 50 μL of Buffer A was added to 50 μL of freshly synthesized amyloids and were mixed by vigorously vortexing the sample to suspend the amyloids in the solution. Carbon coated copper grids with 200 mesh size (EMS, Hattfield USA) were immersed in the sample and allowed to stand for 10 minutes. Excess of sample was blotted away and the grids were immediately flooded with 2% Phosphotungstanic acid. After two minutes, excessive stain was blotted away using a lint-free paper tissue and samples were allowed to air dry. The dried grids were observed using a JEM-2100 transmission electron microscope (JEOL) and the images were recorded with a Erlangshen ES500W camera (Gatan).

### Protein Crystallization Set Up

28–161 protein purified from gel filtration (S200 column attached to ÄKTA system) was used for setting up crystallization trials. For screening of initial crystallization conditions, sitting drop vapour diffusion method was used. The crystallization trial was set up in two-drop, 96-well SwissSci plates (Hampton Research) with various crystallization screening kits Crystal Screen, Index (Hampton Research), Structure Screen (Molecular Dimensions) and Wizard Screen (Emerald Biosystems). Each well contained mixture of 0.7 µL of protein (10 mg/ml) and 0.7 µL of reservoir solution, and was equilibrated with 70 µL of reservoir solution in the well. The plates were sealed with HDclear transparent adhesive sealers (ShurTech Brands LLC., provided by Hampton Research) and incubated at 18 °C maintained in vibration free RUMED unit for crystal growth. Crystallization extension experiments were set-up using the hanging-drop vapour diffusion method in 24 well XLR plates. Crystals were grown by mixing 1 µL of protein (10 mg/ml) and 1 µL of reservoir solution (0.2 M CaCl_2_, 0.1 M Sodium Acetate, pH 5, 20% PEG 6000) on siliconized cover slips (Blue Star, 18 mm) and placed on a well pre-equilibrated with 500 µL of reservoir solution and incubated as mentioned above.

### X-Ray Diffraction Data Collection

Diffraction data of the crystals obtained was collected on an in-house MAR 345dtb image plate detector, mounted on a RIGAKU MicroMax-007 HF rotating anode X-ray generator (λ = 0.15418 nm) operating at 40 kV and 30 mA. The crystal to detector distance was 200 mm. Before data collection the crystals were soaked in cryo-protectant solution containing 20% glycerol mixed with corresponding mother liquor. The temperature during data collection was maintained at 100 K using OXFORD cryostream. Total 120 frames were collected of 10 minutes each with crystal being rotated at the rate of 1°/frame. Images were scaled and merged using HKL2000 suite.

### Structure Refinement

The initial structure for the crystal was obtained through molecular replacement method using the program *MOLREP* from *CCP4* suite using PDB ID 1P8Z as a search model^30^. However, the cell parameters of the crystal under study were different from those of reported earlier, thus the number of chains present in the asymmetric unit were assigned using Matthews Coefficient program of *CCP4* suite of programs^31^. The space group of this crystal was solved by PHASER program using PDB ID 1P8Z^32,33^. The initial models of crystals were refined by rigid body refinement using REFMAC5 which was followed by restrained refinement. Model building and further refinement were done by iterative use of COOT and PHENIX programs till complete models were built^32,34,35^. Addition of solvent molecules present in solution was done when R_work_ value reached around 0.25 in each case. Molecules were added to the electron densities where F_0_ − F_c_ map had a value more than the mean 3σ and the 2F_0_ − F_c_ map showed density at 1σ level, forming at least one hydrogen bond with protein or other solvent.

### Sustained release of protein from β-amyloid assembly

Heat induced amyloids of 28–161 were generated as described above. Starting with a volume of 50 µl, immediately after completion of amyloid formation the vials were centrifuged at 13,000 rpm. 25 μl of supernatant was isolated from the sample and stored separately, and was treated as day 0 sample. The vials were replenished with 25 μl fresh buffer A, then mixed by vortex and were left undisturbed. Samples were collected in similar manner every alternate day and UV-Vis spectroscopy was performed using NanoDrop-2000 (Thermo Fisher Scientific, Wilmington DE, USA). Concentration of protein was recorded at 280 nm, only after subtraction of absorbance of buffer. The concentration of protein was calculated using following equation:

$${c}=({{A}}_{280}\,-\,{{A}}_{320}\,)/\varepsilon $$

where, c is concentration in mg/ml, A_280_ is absorbance recorded at 280 nm post buffer subtraction, A_320_ is absorbance recorded at 320 nm this was chosen as an internal blank for each sample, hence subtracted from A_280_ of each sample, ε represents the molar extinction coefficient (%A). Cumulative release of protein was measured and plotted as a function of time, each data point here represents an average of three independent readings obtained from three independent samples. In case of sustained release in presence of protein stabilizers the protocol was slightly modified. Amyloids were generated and samples were withdrawn as described above, but the replenished buffer A was different. In this case buffer A was separately supplemented with protein stabilizers arginine, sucrose and NaCl, different concentrations were used for each stabilizer. Arginine and Sucrose were supplemented to buffer A in three concentrations 50, 150 and 300 mM, where as NaCl was supplemented in two concentrations 150 and 300 mM as Buffer A already contains 50 mM NaCl, this condition was taken as control. The samples were stored and data was recorded as well as processed as described above.

## Electronic supplementary material

Supplementary Information

## References

[1] Haddad JG, Harper KD, Guoth M, Pietra GG, Sanger JW (1990). Angiopathic consequences of saturating the plasma scavenger system for actin. Proceedings of the National Academy of Sciences of the United States of America.

[2] Lee WM, Galbraith RM (1992). The extracellular actin-scavenger system and actin toxicity. The New England journal of medicine.

[3] Vasconcellos CA, Lind SE (1993). Coordinated inhibition of actin-induced platelet aggregation by plasma gelsolin and vitamin D-binding protein. Blood.

[4] Lind SE, Smith DB, Janmey PA, Stossel TP (1986). Role of plasma gelsolin and the vitamin D-binding protein in clearing actin from the circulation. The Journal of clinical investigation.

[5] Peddada N, Sagar A, Ashish, Garg R (2012). Plasma gelsolin: a general prognostic marker of health. Medical hypotheses.

[6] Peddada N (2013). Global shapes of F-actin depolymerization-competent minimal gelsolins: insight into the role of g2-g3 linker in pH/Ca2 + insensitivity of the first half. The Journal of biological chemistry.

[7] Mounzer KC, Moncure M, Smith YR, Dinubile MJ (1999). Relationship of admission plasma gelsolin levels to clinical outcomes in patients after major trauma. American journal of respiratory and critical care medicine.

[8] Suhler E, Lin W, Yin HL, Lee WM (1997). Decreased plasma gelsolin concentrations in acute liver failure, myocardial infarction, septic shock, and myonecrosis. Critical care medicine.

[9] Wang H (2008). Time course of plasma gelsolin concentrations during severe sepsis in critically ill surgical patients. Crit Care.

[10] Badmalia MD, Singh S, Garg R (2017). & Ashish. Visualizing Temperature Mediated Activation of Gelsolin and Its Deactivation By Pip2: A Saxs Based Study. Scientific reports.

[11] Uversky VN, Li J, Fink AL (2001). Evidence for a partially folded intermediate in alpha-synuclein fibril formation. The Journal of biological chemistry.

[12] Kjaergaard M (2010). Temperature-dependent structural changes in intrinsically disordered proteins: formation of alpha-helices or loss of polyproline II?. Protein science: a publication of the Protein Society.

[13] Arakawa T, Maluf NK (2018). The effects of allantoin, arginine and NaCl on thermal melting and aggregation of ribonuclease, bovine serum albumin and lysozyme. Int J Biol Macromol.

[14] Anderson V, Webb W, Eliezer D (2012). Interplay between desolvation and secondary structure in mediating cosolvent and temperature induced alpha-synuclein aggregation. Physical biology.

[15] Konarev PV, Volkov VV, Sokolova AV, Koch MH, Svergun DI (2003). PRIMUS: a Windows PC-based system for small-angle scattering data analysis. Journal of applied crystallography.

[16] Franke D, Svergun DI (2009). DAMMIF, a program for rapid ab-initio shape determination in small-angle scattering. J Appl Crystallogr.

[17] Krissinel, E. & Henrick, K. Inference of macromolecular assemblies from crystalline state. *J Mol Biol* **372**, 774–797, 10.1016/j.jmb.2007.05.022 (2007). 10.1016/j.jmb.2007.05.022 17681537

[18] Sharma P, Verma N, Singh PK, Korpole S (2016). & Ashish. Characterization of heat induced spherulites of lysozyme reveals new insight on amyloid initiation. Scientific reports.

[19] Fan JS (2017). Structural Basis for pH-mediated Regulation of F-actin Severing by Gelsolin Domain 1. Scientific reports.

[20] Laskowski RA (2009). PDBsum new things. Nucleic acids research.

[21] Nilsson MR (2004). Techniques to study amyloid fibril formation *in vitro*. Methods.

[22] Solomon JP, Page LJ, Balch WE, Kelly JW (2012). Gelsolin amyloidosis: genetics, biochemistry, pathology and possible strategies for therapeutic intervention. Critical reviews in biochemistry and molecular biology.

[23] Srivastava A (2015). Gelsolin Amyloidogenesis Is Effectively Modulated by Curcumin and Emetine Conjugated PLGA Nanoparticles. PloS one.

[24] Van Overbeke W (2015). An ER-directed gelsolin nanobody targets the first step in amyloid formation in a gelsolin amyloidosis mouse model. Human molecular genetics.

[25] Verhelle A (2016). Non-Invasive Imaging of Amyloid Deposits in a Mouse Model of AGel Using (99m)Tc-Modified Nanobodies and SPECT/CT. Molecular imaging and biology: MIB: the official publication of the Academy of Molecular Imaging.

[26] Srivastava A (2018). The Gelsolin Pathogenic D187N Mutant Exhibits Altered Conformational Stability and Forms Amyloidogenic Oligomers. Biochemistry.

[27] Gupta S, Chattopadhyay T, Pal Singh M, Surolia A (2010). Supramolecular insulin assembly II for a sustained treatment of type 1 diabetes mellitus. Proceedings of the National Academy of Sciences of the United States of America.

[28] Puchtler H, Sweat F, Levine M (1962). On the binding of Congo red by amyloid. Journal of Histochemistry & Cytochemistry.

[29] Levine H (1995). Soluble multimeric Alzheimer beta(1–40) pre-amyloid complexes in dilute solution. Neurobiology of aging.

[30] Vagin A, Teplyakov A (1997). MOLREP: an automated program for molecular replacement. Journal of applied crystallography.

[31] Kantardjieff KA, Rupp B (2003). Matthews coefficient probabilities: improved estimates for unit cell contents of proteins, DNA, and protein–nucleic acid complex crystals. Protein Science.

[32] Murshudov GN, Vagin AA, Dodson EJ (1997). Refinement of macromolecular structures by the maximum-likelihood method. Acta crystallographica. Section D, Biological crystallography.

[33] McCoy AJ (2007). Phaser crystallographic software. J Appl Crystallogr.

[34] Emsley P, Cowtan K (2004). Coot: model-building tools for molecular graphics. Acta crystallographica. Section D, Biological crystallography.

[35] Adams PD (2010). PHENIX: a comprehensive Python-based system for macromolecular structure solution. Acta crystallographica. Section D, Biological crystallography.

