# Supplementary material for: Bonsai Gelsolin Survives Heat Induced Denaturation by Forming β-Amyloids which Leach Out Functional Monomer
Source: Sci Rep. 2018 Aug 22;8:12602. doi: 10.1038/s41598-018-30951-3 (PMC6105678; doi:10.1038/s41598-018-30951-3)
Supplement: Supplementary file 1 — Supplementary Information [file 41598_2018_30951_MOESM1_ESM.pdf]

Supplementary Data

BONSAI GELSOLIN SURVIVES HEAT INDUCED DENATURATION BY FORMING β-AMYLOIDS WHICH LEACH OUT FUNCTIONAL MONOMER

MAULIK D. BADMALIA, PANKAJ SHARMA, SHIV PRATAP SINGH YADAV, SHIKHA SINGH, NEERAJ KHATRI, RENU GARG AND ASHISH*

CSIR-INSTITUTE OF MICROBIAL TECHNOLOGY, CHANDIGARH INDIA

**Address correspondence to*: Ashish, PhD. CSIR-Institute of Microbial Technology, Sec 39-A, Chandigarh 160036 INDIA Phone: +172-6665472; Fax: 172-2636680; E-mail: ashgang@imtech.res.in

Figure S1

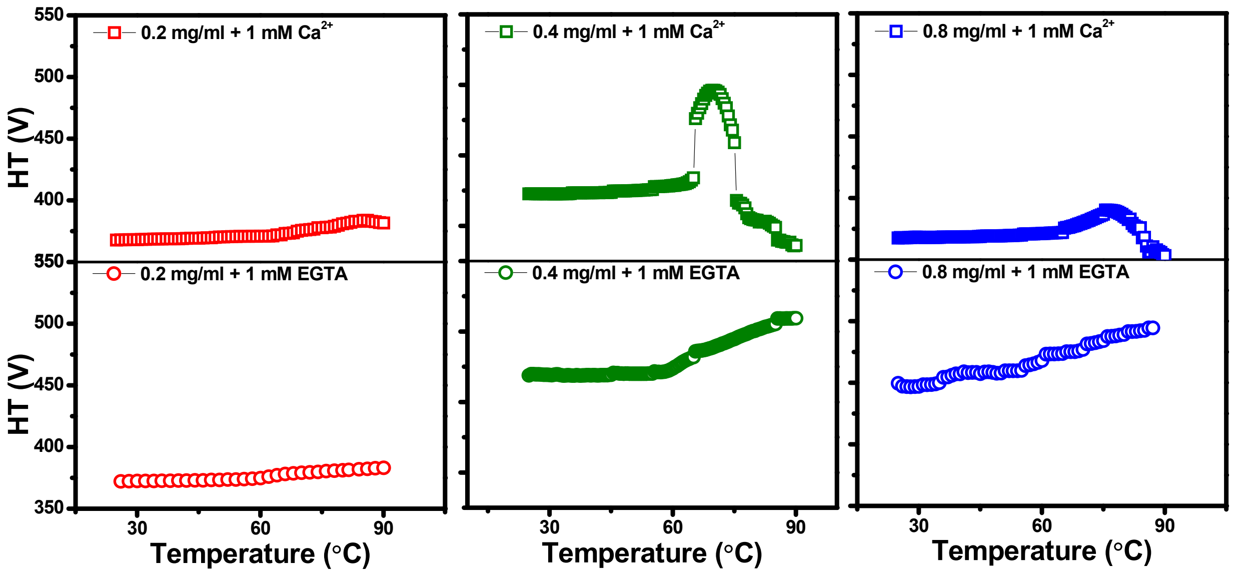

Tracking of HT values recorded during CD experiments have been plotted as a function of temperature. In top mid panel, anomalous increase in the HT values in the sample at 0.4 mg/ml having 1 mM of free Ca^{2+} ions support onset of association in samples. Similar profile was seen in the top right panel but relatively subdued.

Figure S2

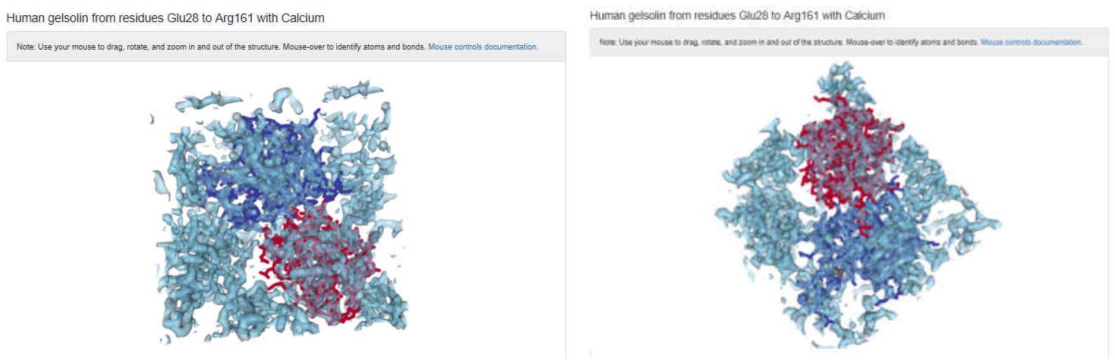

Upper two panels provide rotated views of the electron density maps of the unit cell and the two chains of 28-161 refined inside them.

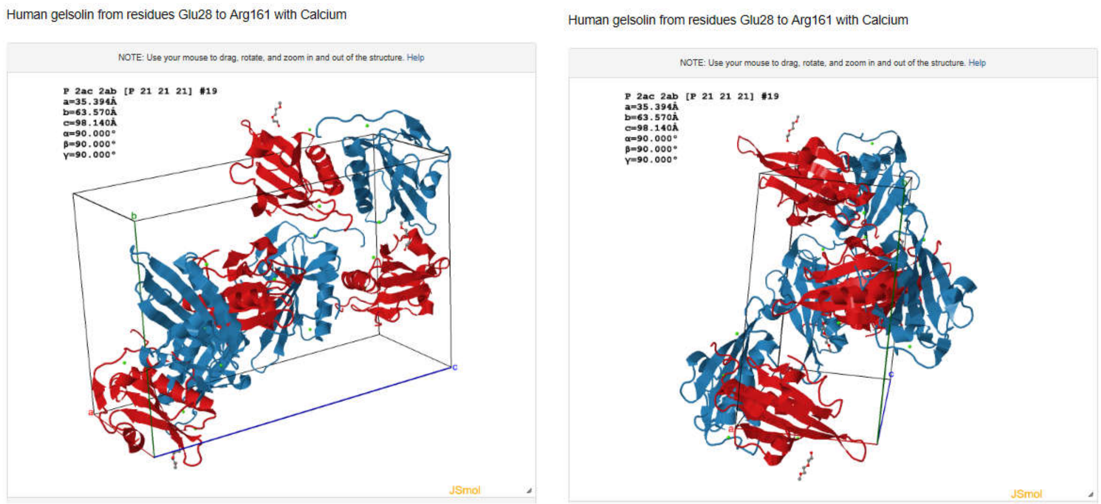

Upper two panels show the arrangement of the chains in the unit cell and their repetitiveness.

Table S1

Different interactions as computed from the web server of PDBePISA have been summarized below. PDB 5DD2 (resubmitted as 5ZZ0) was utilized for the calculations. The blue and red boxes highlight the computed interface area for different relative orientations of chains and complex forming score.

PISA Interface List.

Session Map (id=854-77-AIE)

Start | Interfaces | Interface Search

Monomers

Assemblies

Interfaces in PDB 5dd2 crystal.

Space symmetry group: P 21 21 21. Resolution: 2.60 Å

HUMAN GELSOLIN FROM RESIDUES GLU28 TO ARG161 WITH CALCIUM

Interfaces | XML | View | Details | Download | Search

##			Structure 1				×	Structure 2						interface	ΔG	ΔG	N_{HB}	N_{SB}	N_{DS}	CSS
Id	NN	«»	Range	N_{at}	N_{res}	Surface Å²		Range	Symmetry op-n	Sym.ID	N_{at}	N_{res}	Surface Å²	area, Å²	kcal/mol	P-value				
1	1		G	71	21	6896	◊	A	x,y,z	1_555	69	19	7129	640.6	-5.7	0.357	5	1	0	0.000
2	2		G	56	15	6896	x	G	x-1,y,z	1_455	52	14	6896	507.3	0.1	0.667	4	0	0	0.000
3	3		G	42	8	6896	◊	A	x-1,y,z	1_455	46	12	7129	384.8	2.5	0.831	8	0	0	0.000
4	4		G	38	14	6896	◊	A	-x,y-1/2,-z+1/2	3_545	34	10	7129	308.5	-0.1	0.575	5	0	0	0.000
5	5		A	33	11	7129	◊	G	-x,y-1/2,-z+1/2	3_545	34	13	6896	300.3	-1.3	0.411	0	0	0	0.000
6	6		A	32	10	7129	x	A	x-1/2,-y+1/2,-z+1	4_456	31	9	7129	290.5	-2.8	0.299	3	0	0	0.000
7	7		G	26	8	6896	◊	A	-x+1/2,-y,z-1/2	2_554	26	6	7129	283.1	-1.1	0.537	1	0	0	0.000
8	8		G	25	7	6896	◊	A	-x-1/2,-y,z-1/2	2_454	28	10	7129	238.8	0.5	0.703	5	0	0	0.000
9	9		A	12	2	7129	x	A	-x,y-1/2,-z+1/2	3_545	16	6	7129	116.4	-2.0	0.251	0	0	0	0.000
10	10		G	13	3	6896	◊	A	-x-1,y-1/2,-z+1/2	3_445	13	5	7129	108.9	-0.6	0.539	0	0	0	0.000
11	11		[PG0]G:203	5	1	292	◊	G	x,y,z	1_555	12	5	6896	80.8	2.2	0.240	1	0	0	0.000
12	12		A	9	3	7129	x	A	x-1,y,z	1_455	4	2	7129	49.6	-1.3	0.243	0	0	0	0.000
13	13		[CA]A:202	1	1	85	f	A	x,y,z	1_555	13	8	7129	47.0	-11.1	0.000	0	0	0	0.059
	14		[CA]G:202	1	1	85	f	G	x,y,z	1_555	12	7	6896	46.8	-10.8	0.000	0	0	0	0.059
													Average:	46.9	-10.9	0.000	0	0	0	0.059
14	15		[CA]G:201	1	1	85	f	G	x,y,z	1_555	7	4	6896	41.3	-8.7	0.000	0	0	0	0.045
	16		[CA]A:201	1	1	85	f	A	x,y,z	1_555	6	4	7129	37.6	-7.7	0.000	0	0	0	0.045
													Average:	39.5	-8.2	0.000	0	0	0	0.045
15	17		A	8	2	7129	◊	[CA]A:201	-x,y-1/2,-z+1/2	3_545	1	1	85	28.9	-5.2	0.000	0	0	0	0.000
16	18		G	2	1	6896	x	G	x-1/2,-y+1/2,-z	4_455	5	1	6896	25.2	0.1	0.647	0	0	0	0.000
17	19		G	3	1	6896	◊	[CA]G:202	x-1,y,z	1_455	1	1	85	13.2	-2.1	0.000	0	0	0	0.000
18	20		[PG0]G:203	1	1	292	f	A	-x,y-1/2,-z+1/2	3_545	1	1	7129	1.0	0.0	0.736	0	0	0	0.000

View | Details | Download | Search

PDBe PISA v1.52 [20/10/2014]
